# The Out-Patient Department

**Published:** 1887-05-28

**Authors:** Andrew Clark, Timothy Holmes


### THE OUT-PATIENT DEPARTMENT.

BY SIR ANDREW CLARK, Bart., M.D.; DR. COLLIER; MR. TIMOTHY HOLMES; AND COLONEL MONTEFIORE.

ON Wednesday, the 18th inst., the last of the present series of sessional meetings of The Hospitals Association was held at the Rooms of the Medical Society of London, Chandos Street, W. The chair was taken by Sir Andrew Clark, Bart., M.D., F.R.S., President of the Association. Among those present were Mr. J. E. Aldous, Mr. A. O'D. Bartholeyns, Sec. Middlesex Hospital, the Rev. Wm. Bethel, Miss Blain, Mrs. Bluett, Mr. Henry C. Burdett, Dr. F. D. Drewitt, Miss Franks, Miss F. Goldsmith, Mr. Timothy Holmes, F.R.C.S., of St. George's, Miss Mayne, Miss Meyrick, Mr. P. Michelli, Sec. St. Mary's Hospital, Paddington, Lieut.-Col. Montefiore, Mr. W. H. Pearce, of the Paddington Green Children's Hospital, Mr. A. B. Peirce, Mr. Hy. Potter, F.R.C.S., the Rev. R. J. Simpson, Dr. W. E. Steavenson, Professor Tweedy, F.R.C.S., Miss Louisa Twining, Mr. R. Walford, M.B., Mr. Jas. Wheatley, M.R.C.S., and Miss Wilson. Letters expressing regret at inability to attend were received from Lord Aberdare, Sir T. F. Buxton, Mr. J. H. Buxton, Dr. Theodore Williams, the Rev. H. H. Birley, Dr. Cholmeley, and others.

At the conclusion of the paper on "Hospital Charity," printed in THE HOSPITAL last week, the following discussion took place.

DR. COLLIER.

I have brought forward this subject in as few words as possible, in order that those who have had larger experience may discuss some of the points which I have raised. I should like to add a little to one of the remarks which I have made in the paper I have just read. Speaking as to the prevention of the abuse of medical charity, I say that we should appeal to the leading members of the medical profession. It would perhaps have been better to say, to appeal to those medical men who are connected with our hospitals. I feel sure that if they would take up the matter seriously they can induce the committees of management to prevent the abuse. I believe that the abuse to a great extent lies in the reckless manner in which subscribers distribute their letter of recommendation.

SIR ANDREW CLARK.

The paper which we have just heard deals with a very interesting, a very important, and a very difficult subject; one which has been much discussed in London, and by one or two distinguished persons whom I see before me to-night. I will sum up the points raised by Dr. Collier. His first contention is, that the abuse of hospital charity generally exists, and is great. The second is, that it damages the character of the poor in their thrift. The third is, that it injures greatly the medical profession. The fourth is, that it is quite remediable. The fifth is, that the remedy lies in a more strict and formal inquiry into the cases presenting themselves for relief; and that, as to the last, there are two methods in operation—first, by means of a paid officer; secondly, by means of a branch of the Charity Organisation Society, each case to be determined on its own merits. The author of the paper considers the weight of evidence is against the use of small payments to hospitals, but that he is himself in favour of its trial, in order that the truth may be ascertained. I am particularly anxious to-night to hear some further pronouncements from Mr. Timothy Holmes, who has with so much energy devoted himself to this and to cognate questions.

MR. TIMOTHY HOLMES, F.R.C.S.

You put me, Mr. Chairman, in rather a difficulty, because, although I am profoundly interested in this question, I feel the boring nature of constantly saying the same thing. The view which I take of this question is quite a different one from that of Dr. Collier in his paper, although I perfectly admit the importance of the view which he has taken, and I think I am ready—pretty nearly—to subscribe to every proposition in his paper. I perfectly admit with him the abuse which prevails in the out-patient department, but that which seems to me to be the real, essential element in the matter, and without which we should make no progress whatever towards a radical reform, is this: that it is the medical circumstances of the patient, and not his social condition, to which we should in the first place pay attention. I have had the experience of serving as an out-patient officer for many years, besides which I was for many years also an officer to a dispensary. In both those employments it struck me that the greater part of the patients whom I saw were not susceptible of cure, although they might be of temporary relief by the method of giving them two or three minutes' consultation and a bottle of physic, and telling them to come again. That method of treatment was entirely inapplicable to their diseases, which spring from the neglect of the ordinary rules of hygiene, —morality, prudence, temperance, and everything which people ought to observe who wish to be in health. It is utterly impossible to treat these patients as out-patients, and the attempt to do so is simply a fraud. I mean by a fraud that it is a thing which leads people into a delusion. I appeal to anyone who has been an out-patient officer, and has seen cases of rheumatism, rickets—whatever you like—which were brought on by the neglect of improvidence of every kind. What one wants is some system by which the out-patients shall be really such as advice—which is all that you can give them—will really do something for. That is, in my opinion, the important question in out-patient practice. The question which Dr. Collier treats in this paper is also of importance as regards the social circumstances of the out-patients, but the one involves the other, if you would only persuade hospital authorities to institute a system by which you could have proper patients to deal with. It is quite a mistake to imagine that it is only the poor—the very poor—who are proper subjects for hospital treatment. The rich men, of course, ought to provide themselves with medical attendance: there is no question about that; but there is a large number of patients who are far from being paupers, who are genteel people, who have been perhaps in good circumstances, who are most profoundly in want of hospital treatment, and who are as good claimants as any persons can be. Their social circumstances, if you were to take a line and say, you shall be earning this figure of wages, would mislead you enormously. Patients who have long been in attendance upon a medical man, or at a dispensary, and have derived no good from it, and have often spent as much as they can afford in endeavouring to get themselves treated and cured, and have not got cured—patients, I say, of that kind are surely as much in want of advice as any of the patients who come to see you or any other physician in your consulting rooms, but they differ in not being able to pay the fees. Ought such patients as those to be excluded on the statement of an inquiry officer? It is perfectly monstrous. Patients of that kind should be admitted to what they want, and that is a consultation at the hospital. They ought to be admitted to the hospital far more than the other class I have just been speaking of, who could be very much better treated by medical practitioners who would see them at home. If I may be allowed a little criticism of the paper, I may say that I was sorry to see that there seemed to be so little attempt to deal with this aspect of the question, namely, that the most unsatisfactory part of our out-patient system at the present time is, that so much of it is employed in attempting to do that which it is impossible to do. Great numbers of cases which are admitted are cases which are not hospital cases at all. A man ought not to be picked up out of the streets and taken to a hospital because he has one of the diseases incidental to humanity. Paupers are provided for by the Poor-law authorities. I beg those who are interested in this question to remember that when our hospitals were founded, there was nothing of that kind, and things were then of course very different. There is really no reason for the existence of these enormous out-patient departments in our hospitals; they are perfectly superfluous. One section of those who attended them were provided for by the people, and the other could provide for themselves without any extraordinary demand upon them. I will say a few words with respect to the medical aspect of the case. These great out-patient departments are kept up in a great measure by the authorities of the hospitals for the purpose of providing the hospitals with a selection of proper cases for clinical instruction, and therein I believe the authorities of the hospitals have committed an entire mistake. They have forgotten that old adage, that the half is sometimes better than the whole. They collect a great crowd of cases, and frequently reduce the out-patient department to entire incapacity for clinical treatment. If they had a smaller supply of cases the out-patient officer would have the power of using the out-patient department for clinical instruction very much more to the advantage of the students in many cases. In the relief of the ailments of the poor, in clinical instruction to the students, and in everything for which the out-patient department is used, it would be improved enormously by being restricted, in the first place, to what really can be done, and in the second place by being put under stricter medical rules.

The CHAIRMAN: May I ask you, Mr. Holmes, to explain what you mean by "stricter"?

Mr. HOLMES: Certainly. I do not believe that any real improvement in the out-patient departments will be effected until the principle is adopted, that only a limited number of persons shall be seen in a limited space of time. That is a matter of ordinary common sense. What I mean by stricter medical rules is this: that no person shall be seen in the out-patient department who is not sent there as a proper hospital case by a medical authority. That medical authority might be any medical man residing within a certain limit of the hospital, but at any rate some medical person should see him, and all medical institutions—all those which are really serious medical institutions—should have a right to apply to the hospitals for letters for out-patients. I should say that this plan would involve an entire abrogation of the present system of out-letters. I believe they would be given up without anyone making the slightest objection. The Governors at St. George's gave up the right without the least objection. The applicant should not be entitled to any treatment at all, unless the authority who sent him and the out-patient officer concurred in the opinion that it was a case deserving of medical treatment. That is my opinion. I believe that this abuse will go on, that these enormous numbers will go on, and this farce of medical attendance will go on, until the public will learn that it is perfectly unnecessary; that it is an abuse and an illusion, and must be put a stop to. Then I believe that something of the kind which I have suggested will be adopted.

LIEUT.-COLONEL MONTEFIORE.

I agree very much with the author of the paper. It is a question upon which I feel very strongly. I am told that there is abuse, and when one sees the great numbers of people in the out-patient departments of our hospitals, one cannot help suspecting it. Where there are schools, I have been told that this great crowding is necessary in order that medical men may be able to pick out from the large mass suitable cases for clinical instruction. They come in very large masses. A medical officer makes a selection of those who shall receive tickets for the out-patient department and those who shall be sent away or receive tickets for the casualty department. In that way a selection is made. I believe there is only one hospital where this is done by a student and not by a duly qualified medical man. Then we come to the question of investigation. I think no wage-limit will do, for circumstances may render some suitable cases for gratuitous treatment even where the limit is exceeded. We all know what large sums we have to pay when we have a continuous illness; how heavy are the fees of the medical man; and how we have to look about us to see how to meet these demands. I agree thoroughly with the writer of the paper when he said that the thrift which we are instilling into the ears of poor people is entirely knocked on the head when they find that immediately they are ill they can go to the hospital and get free treatment. They also waste their time in the out-patient departments. Now some of the hospitals have even provided nice little refreshment counters, the out-patient department being turned into a kind of club, where Tommy can have his lolilpop, while his friends have their cups of tea. I believe that a proper system of investigation, after first treatment, is what is wanted, without any wage-limit. Dr. Collier, I think, has made a mistake about the Great Ormond Street Hospital for Children. They did use the Charity Organisation Society in former days, and it was done very strictly, but after some time it lapsed, the authorities of the hospital got tired of it, and now the subscribers to the hospital do not like the plan of inquiry, which no longer goes on. The system of inquiry at the London Hospital is extremely good, but it does not go far enough. Last year they had 80,000 out-patients—attendances, I believe, this means. A small number were cancelled, some on account of their being eligible for Poor-law medical relief, and others because they could afford to pay the outside medical practitioner. But the numbers cancelled were exceedingly small, even compared with the number of inquiries made. I would have the investigation made not only to guard the purse of the hospital, but I would have the inquirer say, "You must go to such and such a dispensary, where you will get relief for a small monthly payment, but you are not eligible for this gratuitous relief." There must be a class between the pauper and those who are able to pay ordinary fees, and these, I think, should be treated at the hospitals. If out-patients are to be seen only for consultative purposes, there is a great chance of this class not being attended to as they deserve.

Mr. HOLMES said he did not mean to restrict the out-patient department solely to consultative purposes.

Lieut.-Col. MONTEFIORE concluded by thanking the author for his important paper.

The CHAIRMAN: Do I understand Lieut.-Col. Montefiore to say that the wage-limit of judging is unsatisfactory?

Lieut.-Col. MONTEFIORE: Yes; that is quite my view. There should, in my opinion, be no wage-limit drawn.

*(To be continued.)*

